# The mental health system in Brazil: Policies and future challenges

**DOI:** 10.1186/1752-4458-2-12

**Published:** 2008-09-05

**Authors:** Mario D Mateus, Jair J Mari, Pedro GG Delgado, Naomar Almeida-Filho, Thomas Barrett, Jeronimo Gerolin, Samuel Goihman, Denise Razzouk, Jorge Rodriguez, Renata Weber, Sergio B Andreoli, Shekhar Saxena

**Affiliations:** 1 Department of Psychiatry, Universidade Federal de São Paulo, São Paulo, Brazil; 2 Centre for Public Mental Health, Health Services and Population Research Department, Institute of Psychiatry, King's College, University of London, London, UK; 3 Division of Mental Health, The Ministry of Health, Brasilia, Brazil; 4 Universidade Federal do Rio de Janeiro, Rio de Janeiro, Brazil; 5 Instituto de Saúde Coletiva, Universidade Federal da Bahia, Salvador, Brazil; 6 Department of Mental Health and Substance Abuse, World Health Organization, Geneva, Switzerland; 7 Center of Evaluation and Data Integration, CAIDI, Universidade Federal de São Paulo, São Paulo, Brazil; 8 Department of Preventive Medicine, Universidade Federal de São Paulo, São Paulo, Brazil; 9 Division of Mental Health, Pan American Health Organization (PAHO), Washington, D.C., USA; 10 Public Health Program, Universidade Católica de Santos, Santos, Brazil; 11 Mental Health: Evidence and Research, Department of Mental Health and Substance Abuse, World Health Organization, Geneva, Switzerland

## Abstract

**Background:**

The aim of this paper is to assess the mental health system in Brazil in relation to the human resources and the services available to the population.

**Methods:**

The World Health Organization Assessment Instrument for Mental Health Systems (WHO AIMS) was recently applied in Brazil. This paper will analyse data on the following sections of the WHO-AIMS: a) mental health services; and b) human resources. In addition, two more national datasets will be used to complete the information provided by the WHO questionnaire: a) the Executive Bureau of the Department of Health (Datasus); and b) the National Register of Health Institutions (CNS).

**Results:**

There are 6003 psychiatrists, 18,763 psychologists, 1985 social workers, 3119 nurses and 3589 occupational therapists working for the Unified Health System (SUS). At primary care level, there are 104,789 doctors, 184, 437 nurses and nurse technicians and 210,887 health agents.

The number of psychiatrists is roughly 5 per 100,000 inhabitants in the Southeast region, and the Northeast region has less than 1 psychiatrist per 100,000 inhabitants. The number of psychiatric nurses is insufficient in all geographical areas, and psychologists outnumber other mental health professionals in all regions of the country. The rate of beds in psychiatric hospitals in the country is 27.17 beds per 100,000 inhabitants. The rate of patients in psychiatric hospitals is 119 per 100,000 inhabitants. The average length of stay in mental hospitals is 65.29 days. In June 2006, there were 848 Community Psychosocial Centers (CAPS) registered in Brazil, a ratio of 0.9 CAPS per 200,000 inhabitants, unequally distributed in the different geographical areas: the Northeast and the North regions having lower figures than the South and Southeast regions.

**Conclusion:**

The country has opted for innovative services and programs, such as the expansion of Psychosocial Community Centers and the Return Home program to deinstitutionalize long-stay patients. However, services are unequally distributed across the regions of the country, and the growth of the elderly population, combined with an existing treatment gap is increasing the burden on mental health care. This gap may get even wider if funding does not increase and mental health services are not expanded in the country. There is not yet a good degree of integration between primary care and the mental health teams working at CAPS level, and it is necessary to train professionals to act as mental health planners and as managers. Research on service organization, policy and mental health systems evaluation are strongly recommended in the country. There are no firm data to show the impact of such policies in terms of community service cost-effectiveness and no tangible indicators to assess the results of these policies.

## Introduction

Brazil is a country of nearly 8.5 million of Km^2 ^and has huge contrasts in its demographic distribution and social indicators. Such a contrast is exemplified by the human development index (HDI): one of the biggest economies in the world according to GDP, Brazil is ranked 79th according to its HDI and is classified as a low middle income country by the World Bank[1]. Brazil has a population of 188 million people (July 2006 est.), with 68.1% between 15 and 64 years of age, 25.8% 0–14 and 6.1% 65 years old and over (IBGE 2006). Portuguese is the official language and the main ethnic groups are White (53.7%), Mulatto (mixed white and black, 38.5%) and Black (6.2%). The remaining 0.9% of the population is comprised of Japanese, Arab, Amerindian and others. The majority of the population is Roman Catholic (73.6%), with the other important religions being Protestant (15.4%), Spiritualist (1.3%) and Santeria (0.3%). The unemployment rate is fairly high and 22% of the population lives below the poverty line[2]. The total health expenditure per capita is 597 US$ and the proportion of the health budget to GDP is 7.6%. Of the total adult population, 0.7% has HIV/AIDS (660,000 people). The Infant Mortality Rate is 22.5 deaths per 1,000 births[3].

All Brazilian citizens have a constitutional right to health care provided by the state, free of charge, with no discrimination of any kind. The Unified Health System (SUS) is a single, public system to aggregate all health services provided by federal, state and municipal public institutions through direct and indirect administration, as well as foundations supported by public authorities. The private sector is also allowed to be part of the system under contract, although public authorities retain the power to govern, control and inspect the services provided. The Ministry of Health is responsible for monitoring and directing all activities related to health, including medical care[4]. The hierarchy should operate on referral and counter-referral mechanisms, from the least to the most complex level of care, ensuring the continuity of care by means of the primary caregiver. Provision of health services is the responsibility of municipal governments, financially aided by the federal government and the states.

The Brazilian Mental Health Policy is essentially based on the Caracas Declaration[5,6], the four main points of which are described as follows: a) to guarantee civil rights for people with mental disorders according the Principles for the Protection of Persons With Mental Illness and the Improvement of Mental Health Care – UN General Assembly Resolution 46/119 of 17 December 1991[7]; b) to decentralize psychiatric care; c) to protect patients under treatment in the existing hospitals; and d) to develop a diverse network to provide access, efficacy and efficiency for people with mental disorders. The Ministry of Health of Brazil supported the project for a Mental Health Law, which essentially proposes the progressive substitution of psychiatric beds for Community Social Psychiatric Centres, labelled "Centros de Atenção Psicosocial-CAPS"[8]. In addition, the law introduced a program to pay a monthly bonus to the families of long-stay patients, to encourage long-stay patients to leave hospital and return to the community[9]. The main aim of this paper is to assess the mental health system of the country by focusing on its human resources and the availability of mental health services.

## Methods

The World Health Organization Assessment Instrument for Mental Health Systems (WHOAIMS) has been applied in Brazil and this paper will focus on the sections of Mental Health Services and Human Resources. In addition, the information collected in this survey will be based on two main sources: a) the Executive Bureau of the Department of Health (Datasus); and b) the National Register of Health Institutions (CNES).

*World Health Organization Assessment Instrument for Mental Health Systems (WHOAIMS)*[10] is a questionnaire developed by the World Health Organization to gather information from mental health systems in countries or regions. The WHO AIMS comprises 155 items divided into six domains: 1) Policy and Legislative Framework; 2) Mental Health Services; 3) Primary Mental Health Care; 4) Human Resources; 5) Public Education and Link with Other Sectors; and 6) Monitoring and Research.

*The Executive Bureau of the Department of Health (Datasus) *compiles the data drawn from the Hospital Information System of the Unified Health System (SIH/SUS)[11,12] and the data from the Outpatient Information System – SIA/SUS. These two datasets are referred as "Datasus" and have been available online since July 1994.

*The National Register of Health Institutions (CNES)*[13] is a national record of all Health Units linked to the Unified Health System, which comprises information about physical area, human resources, the availability of medical equipment, the number of outpatient attendances and the number of hospital admissions. The CNES information is available on-line together with the files generated monthly by DATASUS. The CNS files do not hold personal data, preventing any estimation of the prevalence or incidence of mental disorders.

## Results

### Human resources

As can be seen in Table 1, there are 6003 psychiatrists, 18763 psychologists, 1985 social workers, 3119 nurses and 3589 occupational therapists working for the Unified Health System. Overall, human resources are not sufficient and are unequally distributed in the different geographical areas of the country, as shown in Table 2, for data collected for the year 2005. The number of psychiatrists is approximately 5 per 100,000 inhabitants in the Southeast region, and in the Northeast region there is less than 1 psychiatrist per 100,000 inhabitants. The number of psychiatric nurses is insufficient in all geographical areas of the country. Psychologists outnumber psychiatrists in all regions of the country. The distribution of human resources between urban and rural areas is also disproportionate. The density of psychiatrists in or around the largest city (Metropolitan São Paulo) is 1.75 times greater than the national average.

**Table 1:** The total number of workers in mental health facilities and private practices per 100,000 inhabitants, Brazil, 2005.

**Worker**	**Number**	**Rate per 100,000 inhabitants**
1. Psychiatrists^1^	**6003**	3.26
2. Other medical doctors, not specialized in psychiatry^2^	**1065**	0.58
3. Nurses	**3119**	1.69
4. Psychologists^3^	**18763**	10.19
5. Social workers^4^	**1985**	1.08
6. Occupational therapists^5^	**3589**	1.95

TOTAL	34524	18.74

Source: CNES, 2005.

^1 ^Psychiatrists in private or public health services; professionals that work only in private practices were not included. Source: tabwin-CNES, December 2005.

^2 ^The total number of clinicians and nurses working in mental health is not available, but at least 1065 other medical doctors (not specialized in psychiatry) and 3119 nurses are registered in mental health services. In all the services where there are other areas of health care, such as in general hospitals or outpatient services, it is not possible to identify how many doctors or nurses, besides the mental health team, also work with mental health care.

^3 ^Psychologists in private or public health services. Source: tabwin-CNES, December 2005. The number of psychologists in health services does not include those working in private offices. The Federal Psychology Board had records of **125,397 **registered psychologists in the country in 2005; 41% of them worked in private practices (full time or part time); 11% worked in companies; and 10% worked in schools (IBOPE, 2004).

^4 ^There are 14,338 social workers in health services. We do not have the number of social workers working exclusively with mental health, but 949 work in psychiatric hospitals and in CAPS, and 1036 work in other outpatient mental health services (source CNES – TABWIN, Dec 2005).

^5 ^Occupational therapists in private or public health services; therapists working only in private practices were not included. Source: tabwin-CNES, December 2005.

No estimates are available of the number of other health or mental health workers (including assistants, non-doctor/non-physician primary health care workers, health assistants, medical assistants, professional and paraprofessional psychosocial counselors).

**Table 2:** The Rates per 10,000 inhabitants for human resources and mental health services, Brazil, 2005

**Geographical Regions**	**Psychiatrists**		**Psychologists**		**Social workers**		**Nurses**	
	
	**N**	**rate**	**N**	**Rate**	**N**	**rate**	**N**	**Rate**
**North**	93	0.063	600	0.408	844	0.570	10	0.007
**Northeast**	948	0.186	2996	0.587	3543	0.690	218	0.043
**Center-West**	312	0.240	1623	1.246	932	0.710	36	0.028
**Southeast**	3570	0.455	10117	1.289	7069	0.900	329	0.042
**South**	1080	0.400	3427	1.271	1950	0.720	87	0.032
**Brazil**	6003	0.326	18763	1.019	14338	0.720	680	0.037

**Source: **CNES, 2005

### Psychiatric hospitals, community-based psychiatric inpatient units and residential facilities

Table 3 displays the number of beds distributed among the psychiatric hospitals and psychiatric wards in general hospitals, the number of psychiatric beds in general hospitals, sheltered homes and custody-psychiatric hospitals. Brazil has 228 psychiatric hospitals that are either public or that provide services to the public system, and which offer a total of 42,076 beds (22.84 beds per 100,000 inhabitants). Another 4855 private beds are available in psychiatric hospitals that accept private medical insurance payments and 3,114 private beds available in at least 50 fully private psychiatric hospitals (Data retrieved for the National Registry of Health Facilities (CNES) in June 2006. Therefore, the total number of beds in psychiatric hospitals in the country is 50,045 (27.17 beds per 100,000 inhabitants). The rate of patients in psychiatric hospitals is 119 per 100.000 inhabitants (the occupancy rate in mental hospitals is 93%). The average length of stay in mental hospitals is 65.29 days. In Brazil, 105 community-based psychiatric inpatient units provide 2074 beds (1.13 beds per 100,000 inhabitants). Patient mean length of stay was 14.02 days per discharge. Moreover, 592 general hospitals offer some psychiatric beds in general wards or emergency-care back-ups (n = 1224 beds, with 1094 being linked to SUS and 130 in private institutions). In addition to beds in mental health facilities, there are also 3677 beds for persons with mental disorders in forensic inpatient units. Brazil has 25 hospitals for custody and psychiatric treatment, which are psychiatric hospitals kept by the prison system for the exclusive treatment of patients that committed a crime and are suspected of having or actually have a mental disorder that requires specialized treatment.

**Table 3:** The number of psychiatric beds in psychiatric and general hospitals (June 2006)

	N	Number of beds	Beds/10000 inhab.	Admissions	Mean length of stay in days
Psychiatric wards in general hospitals	105	2074	1,13	46650**	14
Psychiatric beds in general hospitals	592	1224	0,66		NA
Sheltered Homes	418	3344	1,81	2115	NA
Psychiatric Hospitals	228	50045	27,17	218544	65,29***
Custody-Psychiatric Hospitals	25	3677*	2,0	3677	NA

**TOTAL**	1418	56687	32,77	224336	

* Estimated number from number admitted

** Admissions from general hospital psychiatric wards plus psychiatric beds in general hospitals.

*** solely for the 42,076 state beds.

**Source: **DATASUS,2006; CNES, 2006

In 2006, there were 418 community residential facilities in the country. Each residence may have up to 8 residents and, together, these residences provide around 3800 places (2.06 places per 100,000 inhabitants). The number of patients in community residential facilities in 2006 was 2480. The number of beds in other residential facilities in the country, such as in homes for persons with mental retardation, in detoxification inpatient facilities and in homes for the destitute, is unknown. Regarding the rural/urban density of psychiatric beds: the 16 largest cities in the country have 22,026 psychiatric beds for 63,064,612 inhabitants, giving a rate of 36.8 beds per 100,000 inhabitants, a density 1.36 times higher than the national average. However, the density of psychiatric beds in or around the largest city is 0.53 times the density of beds in the entire country, showing the imbalance of the system where some areas can be more affected by the shortage of beds than others.

### Outpatient mental health facilities

The Psychosocial Community Centers (CAPS) provide day hospital care, which is considered intensive care. They were developed for treating severe mental disorders and are classified according to three degrees: complexity, population covered, and funds allocated. In June 2006, there were 848 CAPS registered in Brazil: 673 for adults, 109 for problems related to alcohol and drug use, 66 of which are for children and adolescents only. As can be seen in Table 4, there are 355 CAPS I, 290 CAPSII and 28 CAPSIII. CAPS I are units that open 5 days a week and are based in small towns with the population sizes between 20,000 and 50,000 inhabitants (19% of Brazilian cities where 17% of the population live). The CAPSII are intermediate units meant to attend medium size cities with more than 50,000 inhabitants (10% of the cities in Brazil, home to around 65% of the population). The CAPSIII are large units which are meant to be opened 24 hours a day, including 5 24 hour beds for admissions. These services target large cities with more than 200,000 inhabitants (around 2.5% of Brazilian cities, covering 43% of the population). The CAPS for alcohol and drugs are intended for cities with more than 200,000 inhabitants, border sites and/or cities where epidemiological findings show a high prevalence of alcohol and drug abuse. There are, therefore, around 0.9 CAPS per 200,000 inhabitants, but they are also very unequally distributed, with the Northeast and the North regions presenting lower figures than the South and Southeast regions.

**Table 4:** The number of psychosocial community centers (CAPS) in Brazil, by modality, in June 2006

MODALITY	NUMBER	CHARACTERISTICS
CAPS I	355	Minimum team members: 1 psychiatrist or physician specialized in mental health 1 registered nurse 3 professionals qualified in other areas: psychologist, social worker, occupational therapist, pedagogue or other professionals needed for the therapy plan 4 technicians: nursing assistant or technician, administrative technician, educational technician, and artisan.

CAPS II	290	Minimum team members: 1 psychiatrist 1 registered nurse specialized in mental health 4 professionals qualified in other areas: psychologist, social worker, occupational therapist, pedagogue, physical education teacher, or other professionals needed for the therapy plan 6 technicians: nursing assistant or technician, administrative technician, educational technician, and artisan.

CAPS III	28	Minimum team members: 2 psychiatrists 1 registered nurse specialized in mental health 5 professionals qualified in other areas: psychologist, social worker, occupational therapist, pedagogue, or other professionals needed for the therapeutic plan 8 technicians: nursing assistant or technician, administrative technician, educational technician, and artisan. Provides crisis services with priority to patients participating in one of the therapy projects in one of the CAPS, for a maximum of 7 days in a row or a total of 10 days in 30 days.

CAPSi	66	Provides services for infant psychiatry Team: 1 psychiatrist or neurologist or pediatrician specialized in mental health

CAPSad	109	Treatment of problems associated with the consumption of alcohol and other drugs

TOTAL	848	

Source: National Division of Mental Health, Ministry of Health.

### The return home program

The return home program aims to effectively contribute to the process of social inclusion of individuals with long histories of hospitalization in psychiatric hospitals through a monthly rehabilitation benefit (equivalent to about US$ 140) paid directly to patients when they leave psychiatric hospital. This financial benefit is transferred to the patient's own bank account. It strongly enhances the emancipation of individuals with mental disorders and the processes of deinstitutionalization and reduction of long-stay psychiatric beds in states and municipalities. In 2006 there were 2519 people receiving the benefit in the country.

### Mental health in primary health care

The Brazilian federation is comprised of 26 states, one federal district and 5560 cities. In 2005, there were 39,824 primary care units (PHC) and 25,141 physicians working in the Family Program Teams (PSF), which include at least one nurse and one health agent each, leading to a total number of 104,789 doctors, 184,437 nurses and nurse technicians and 210,887 health agents working at the primary care level. Most primary health care (PHC) services have at least one physician on site or available for referrals, and only in the most distant regions are there non-physician based PHC clinics.

## Discussion and recommendations

### Human resources

There are 5259 psychiatrists, 12377 psychologists, 11958 social workers, 3119 nurses and 2661 occupational therapists working for the Unified Health System. The number of psychiatrists is still far from ideal: they are concentrated in the wealthier areas of the country. There is a shortage of infant psychiatrists and of specialized training in areas such as eating disorders, elderly psychiatry and forensic psychiatry. Most of the psychiatrists and specialized mental health workers are concentrated in the capitals and training is rarely carried out at the new services, i.e., at the Psychosocial Community Centers level. The number of psychologists is higher than the number of other mental health professionals for all regions of the country and it is mandatory to expand training and to incorporate more psychiatric nurses to work at community level[14].

### The role of the CAPS system

The CAPS units have become the cornerstone of the Brazilian Psychiatric Reform[15]. These community services are responsible for treating severe mental disorders and to articulate the liaison with primary care units to coordinate psychiatric care in a defined catchment area. The matrix of mental health care should be centralized at CAPS level, where many actions should take place, such as supervision and brief training of mental health workers and involving families and patients to combat stigma. CAPS should also be the main referral for severe cases, averting in-ward admissions (psychiatric hospitals or psychiatric wards in general hospitals) wherever possible. This mental health care matrix (crisis intervention, return home program, promoting civilian rights of users, combating stigma), where all activities are centralized at CAPS level, is very important to replace the anachronistic custodial model.

By the end of 2001, there were 295 CAPS in the country and in June 2006 there were 848[16], though concentrated in the South and Southeast regions. The large centres where the epidemiological findings showed that high levels of violence and/or a fast deinstitutionalization were occurring received implementation priority. It is acknowledged, however, that the overrepresentation of the CAPS system in the wealthiest regions of the country is still related to existing geographical inequities.

### Treatment of severe cases

The number of psychiatric beds is declining, and many acute cases have now been treated in general hospitals, and in the community. The purpose of CAPS is both to provide for the various needs of patients with serious mental disorders and to act in coordination with the primary care services to treat those mental disorders that are most prevalent among the population. It is clear that the number of units is far from the ideal number needed in the country: around 1 unit per 200,000 inhabitants. There are only 66 CAPS directed to infant psychiatry, a number fairly low for the needs of the population. The number of psychiatric beds in general hospitals increased in the nineties[17], but very few units have been opened in recent years, though the long experience of the country in this area[18,19]. The length of stay in these units tends to be much shorter, the stigma attached to admission is less pronounced and associated medical conditions can be diagnosed.

It is noteworthy that some patients with severe mental disorders, particularly Intellectual Disabilities, go untreated in Brazil[20,21]. Of those receiving treatment, most are treated with conventional anti-psychotic agents, afforded by the essential list of medicines, although in the most developed sites the number of patients with access to the new generation of antipsychotics is increasing steadily. Studies on coverage, i.e., the percentage of patients who really benefit from both conventional and new generation antipsychotics are still missing in the country. In the less privileged areas it is important to improve access to treatment by means of the conventional medicines available in the SUS. It would be advisable to improve coverage of treatment, i.e., to evaluate the effectiveness of dispensation of anti-psychotic medication by general community health workers, those involved in the PSF program and the PHC units, so as to reduce relapse and admission rates of severe cases.

### Return home program

Regarding the return home program, until 2006, 2519 patients were already located, but no long-term studies on the effectiveness of this program have been conducted. Patients transferred to the community may suffer an increased risk of suicide[22] and some long stay patients present significant impaired social functioning and lack of autonomy[23]. There are many ongoing long-stay patients to be transferred and it is necessary to have longitudinal data to assess the cost and efficacy of the program.

### Primary health care

It is important to develop mental health guidelines for primary care health professionals and to train health workers acting in primary care. In particular, the training could be intensified for health teams (general physician, nurse and health agents) acting in the PSF program[24] spread across the country. It is also important to strengthen the links between the PSF and the PHC teams and the CAPS mental health workers in the community. Training alone is not sufficient to guarantee sustainability of actions so it is important to develop long term follow-ups of teams. Health agents could play an important role in the delivery of psychotropics for maintaining adherence in severe mental disorders. This interaction between primary care health workers and the mental health teams working at CAPS level should facilitate referrals to specialized psychiatric treatments. The development of key common mental disorders guidelines to be applied in primary health care should be developed by universities and provided by the National Division of Mental Health of the Ministry of Health.

### Epidemiological tools

The Division of Mental Health should develop standardized epidemiological tools and supervise mental health services in data collection for CAPS, day-hospitals, psychiatric wards in general hospitals and out-patient units. It would be important to have indicators such as the number of new cases seen in the systems, the number of persons being reached, and so forth. These epidemiological instruments can be developed by universities and be available to all mental health services in the country. This information could be gathered at the National Division of Mental Health and linked to the DATASUS system. The available data provided by the CNS files are concerned rather more with the number of attendances than with providing statistics for estimating prevalence and incidence of mental disorders.

### The call for action in Brazil

Mental disorders are associated with significant negative consequences that affect society as a whole. The economic and social impact of mental disorders is observed in terms of human capital losses, the reduction of qualified and educated manpower, impairment of the health and global development of children, workforce losses, violence, criminality, homelessness and poverty, premature death, health impairment, unemployment and out-of-pocket costs for family members[25-27]. The adoption of economic criteria for resource allocation focused to treat mental disorders should be based on cost-effectiveness data for interventions and services and evaluation of broad outcomes, prioritizing the needs of those people suffering from the most burdensome mental disorders and targeting vulnerable and disadvantaged groups[27,28].

The WHO report, New Understanding, New Hope[29], and the Lancet Global Mental Health call followed by the launch of the mental health call movement[30], stress the negligence and stigma around mental illness as main factors impeding the mental health field from being adopted as a public health priority, particularly in low and low middle income countries[31]. This call was also heard in Brazil where more than 400 mental health professionals gathered to assess current mental health policies and to raise recommendations on how to scale up services in the country. Further to the consensus that mental health should be placed on the public health priority agenda, the main recommendations that rose during the discussions and presentations were as follows: a) to increase the expenditure on mental health, currently at 2.5% to 5.0% of the total health budget; b) to carry on the expansion of Community Centers (n = 1086); c) to expand the number of psychiatric beds in general hospitals (n = 2074); d) to increase transference of patients from long-stay psychiatric hospitals to community residential facilities (n = 2480); e) to expand mental health training for general physicians and primary care health professionals; f) to improve the mental health information system in the country; g) to develop a Master of Science in Mental Health Planning at university level with financial support from the Ministry of Health; and h) to apply an extra three million dollars to priority mental health research, combining financing from the Ministry of Health and the Brazilian Research Council (CNPq).

The country has opted for innovative services and programs, such as the CAPS and the development of a primary care network, and the Return Home program. In little more than a decade, hundreds of services were established in the whole country, and older outpatient services and day hospital programs were remodelled according to general guidelines to maximize limited resources by adopting a mixed system of mental health outpatient services, day hospitals and therapy workshops[32]. However, services are unequally distributed across the country's regions, and the growth of the elderly population is increasing the burden[33], combined with an existing treatment gap in mental health care[34,35]. This gap may get even wider if funding does not increase and mental health services are not expanded in the country[36]. There is no solid data to show the impact of such policies would have in terms of community services cost-effectiveness and no tangible indicators to assess the results of these policies. Despite some acknowledged advances, many hurdles have to be overcome before the country achieves a solid and sustainable psychiatric care system.

## Competing interests

The authors declare that they have no competing interests.

## Authors' contributions

MDM designed the project, collected data, and drafted the manuscript. JJM, SBA and NA–F designed the study, supervised the field-work, and made contributions to the main manuscript. PGD and RW were responsible for gathering the data in the Division of Mental health of the Ministry of Health, and gave substantial contributions to the draft of the paper. TB, JR, and SS were involved in the development of the WHO-AIMS questionnaire adopted in the study. JG, DR and SG did collect data from the main databases of the Ministry of Health, and participated in the analysis and interpretation of the findings. All authors read and approved the final manuscript.
